# Ginsenoside Rh2 inhibits hepatocellular carcinoma through β-catenin and autophagy

**DOI:** 10.1038/srep19383

**Published:** 2016-01-19

**Authors:** Zhiqing Yang, Tingting Zhao, Hongli Liu, Leida Zhang

**Affiliations:** 1 Institute of Hepatobiliary Surgery, Southwest Hospital, Third Military Medical University, Chongqing, China; 2 Institute of Immunology, Third Military Medical University, Chongqing, China

## Abstract

Hepatocellular carcinoma (HCC) is the most common liver cancer, with a very poor prognosis. There is an urgent need for an effective therapy for HCC. Ginsenoside Rh2 (GRh2) has been shown to significantly inhibit growth of some types of cancer, whereas its effects on HCC have not been examined. Here, we treated human HCC cells with different doses of GRh2, and found that GRh2 dose-dependently reduced HCC viability, in either CCK-8 assay or MTT assay. The effects of GRh2 on the cancer stem cells (CSCs)-like cells were determined by aldefluor flow cytometry and by tumor sphere formation, showing that GRh2 dose-dependently decreased the number of these CSCs-like cells in HCC. Autophagy-associated protein and β-catenin level were measured in GRh2-treated HCC cells by Western blot, showing that GRh2 increased autophagy and inhibited β-catenin signaling. Expression of short hairpin small interfering RNA (shRNA) for Atg7 in HCC cells completely abolished the effects of GRh2 on β-catenin and cell viability, while overexpression of β-catenin abolished the effects of GRh2 on autophagy and cell viability. Together, our data suggest that GRh2 may inhibit HCC cell growth, possibly through a coordinated autophagy and β-catenin signaling.

Hepatocellular carcinoma (HCC) is the most common liver cancer, and has a poor therapeutic outcome after combined surgical treatment, radiotherapy and chemotherapy^1^,^2^,^3^. Although the comprehension of the HCC carcinogenesis has significantly improved in the past years, the prognosis of HCC remains poor. Hence, there is an urgent need for developing effective therapies for treating HCC.

Cancer stem cells (CSCs) are cancer cells similar to normal stem cells, and are believed to be responsible for cancer relapse and metastasis after primary tumor resection^4^,^5^. Recently, it is believed that removal of CSCs is critical for an effective cancer therapy. Thus, identification of CSCs in a particular cancer becomes very important. To date, different cell surface proteins, e.g. CD133, have been used to isolate CSCs from a variety of cancers, including HCC^6^,^7^,^8^. Moreover, high aldehyde dehydrogenase (ALDH) activity has also been used to identify CSCs, using an aldefluor assay, specifically for CSCs in HCC^9^. Of note, all current approaches using any surface markers or combinations are not able to purify 100% CSCs, but simply enrich the population that contain CSCs. Hence, those isolated CSCs by surface markers may be called as CSCs-like cells.

Autophagy is a catabolic pathway to degrade and recycle cellular compartments for cell survival at nutrient deprivation on physiological cellular metabolism, whereas it often leads to cell death^10^. Moreover, autophagy also plays a critical role in tumor, since it significantly reduces tumor growth^11^,^12^. Microtubule-associated protein 1A/1B-light chain 3 (LC3) is a soluble cellular protein. During autophagy, autophagosomes engulf cytoplasmic components, resulting in conjugation of a cytosolic form of LC3 (LC3-I) to phosphatidylethanolamine to form LC3-phosphatidylethanolamine conjugate (LC3-II). Thus, the ratio of LC3-II to LC3-I represents the autophagic activity^10^,^11^,^12^. Beclin and Atg7 are two other autophagy-associated proteins^13^. Wnt/β-catenin signaling has been shown to play a role in autophagy^14^,^15^,^16^,^17^,^18^,^19^. In the canonical Wnt pathway, the binding of a Wnt-protein ligand to a Frizzled family receptor activates β-catenin, and its nuclear translocation and retention lead to regulation of gene transcription^20^.

Ginsenoside Rh2 (GRh2) is a well-characterized component in red ginseng, and has been shown of therapeutic effects on inflammation^21^ and some cancers^22^,^23^,^24^,^25^,^26^,^27^,^28^,^29^,^30^,^31^, although the underlying mechanisms are largely unknown. However, whether GRh2 may be an effective treatment for HCC has not been investigated.

Here, we treated human HCC cells with different doses of GRh2, and found that GRh2 dose-dependently reduced HCC viability, in either CCK-8 assay or MTT assay. The effects of GRh2 on the CSCs-like cells were determined by aldefluor flow cytometry and by tumor sphere formation, showing that GRh2 dose-dependently decreased the number of these CSCs-like cells in HCC. Autophagy-associated protein and β-catenin level were measured in GRh2-treated HCC cells by Western blot, showing that GRh2 increased autophagy and inhibited β-catenin signaling. Expression of short hairpin small interfering RNA (shRNA) for Atg7 in HCC cells completely abolished the effects of GRh2 on β-catenin and cell viability, while overexpression of β-catenin abolished the effects of GRh2 on autophagy and cell viability. Together, our data suggest that GRh2 may inhibit HCC cell growth, possibly through a coordinated autophagy and β-catenin signaling.

## Materials and Methods

### Experimental protocol approval

All experimental protocols were approved by the Research Bureau of Third Military Medical University. All the animal protocols were approved by the IACUC of Third Military Medical University.

### HCC cell line and GRh2 *in vitro* administration

HepG2 and Huh7 are two human HCC cell lines, which were purchased from American Type Culture Collection (ATCC, Rockville, MD, USA), and cultured in Dulbecco’s modified Eagle’s medium (DMEM, Invitrogen, Carlsbad, CA, USA) supplemented with 15% fetal bovine serum (FBS; Sigma-Aldrich, St Louis, MO, USA) in a humidified chamber with 5% CO_2_ at 37 °C. GRh2 (Weikeqi Bioscience, China) was prepared in a stock of 100 mg/ml and applied to cultured GBM cells at 0.01 mg/ml, 0.1 mg/ml and 1 mg/ml, respectively .

### Lentivirus production

Plasmids carrying luciferase reporter under a CMV promoter (CMVp-luciferase) were purchased from Clontech (Mountain View, CA, USA). The coding sequence of human β-catenin was amplified using human liver cDNA as a template, and cloned into pLVX-ZsGreen1-C1 vector (Clontech). The shRNA for autophagy-related protein 7 (Atg7) was purchased from Qiagen (Hilden, Germany). A scramble sequence was used as the mock control (scr). Human shATG7 target sequence: 5′-GCCTGCTGAGGAGCTCTCCAT-3′; scr sequence: 5′-CTGCGATGCGCGTTCCGCTTA-3′. In the scope of the current study, we did not detect the effects of scr on our results, compared to untreated cells. Thus, only data from scr-treated cells were shown in the figures. HEK293T cells (NIH, Bethesda, MA, USA) were used for production of CMVp-luciferase lentiviral particles. HEK293T cells were seeded in a 100 mm dish at 50,000 cells/cm^2^ and co-transfected with 10 μg of recombinant DNA plasmids and 5 μg each of packaging plasmids (REV, pMDL and VSV-G) using Lipofectamine-2000 (Invitrogen). The supernatant containing lentiviral particles was collected 48 hours after transfection and filtered through a 0.45 μm syringe filter.

### Lentivirus transduction of HCC cells

The HepG2 cells were seeded in 100 mm plates at 15,000 cells/cm^2^ one day prior to lentiviral infection. The lentiviral particles were added along with 10 μg/ml polybrene (Sigma-Aldrich) to the cell culture for 24 hours. Infected cells were selected by ampicillin resistance.

### Cell transfection

Transfection of HCC cells with either shAtg7, or β-catenin-expressing plasmids, or control plasmids was performed with Lipofectamine-2000 (Invitrogen), and had a nearly 100% transfection efficiency based GFP expression on the transfected cells.

### Aldefluor analysis

The implanted tumor was digested with 10 μg/ml Trypsin (Sigma-Aldrich) for 25 minutes to prepare single cell fraction for flow cytometry. Cells in culture were dissociated with 10 μg/ml Trypsin for 2 minutes to prepare single cell fraction for flow cytometry. The Aldefluor Kit (StemCell Technologies, China) was applied according to the manufacturer’s instructions, to identify high ALDH enzymatic activity. Flow cytometry was performed using a FACSAria (Becton-Dickinson Biosciences, San Jose, CA, USA) flow cytometer.

### MTT assay

For assay of cell viability, cells were seeded into 24 well-plate at 10000 cells per well and subjected to a Cell Proliferation Kit (MTT, Roche, Indianapolis, IN, USA), according to the instruction of the manufacturer. The MTT assay is a colorimetric assay for assessing viable cell number, taking advantage that NADPH-dependent cellular oxidoreductase enzymes in viable cells reduce the tetrazolium dye 3-(4,5-dimethylthiazol-2-yl)-2,5-diphenyltetrazolium bromide (MTT) to its insoluble formazan in purple readily being quantified by absorbance value (OD) at 570 nm. Experiments were performed 5 times.

### Cell counting kit-8 (CCK-8) assay

The CCK-8 detection kit (Sigma-Aldrich) was used to measure cell viability according to the manufacturer’s instructions. Briefly, cells were seeded in a 96-well microplate at a density of 5 × 10^4^/ml. After 24 h, cells were treated with resveratrol. Subsequently, CCK-8 solution (20 ml/well) was added and the plate was incubated at 37 °C for 2 h. The viable cells were counted by absorbance measurements with a monochromator microplate reader at a wavelength of 450 nm. The optical density value was reported as the percentage of cell viability in relation to the control group (set as 100%).

### Primary Tumor Sphere Culture

Purified tumor cells by flow cytometry were washed, acutely dissociated in oxygenated artificial cerebrospinal fluid and subject to enzymatic dissociation. Tumor cells were then resuspended in tumor sphere media (TSM) consisting of a serum-free DMEM, human recombinant EGF (20 ng/ml; Sigma-Aldrich), bFGF (20 ng/ml; Sigma-Aldrich), leukemia inhibitory factor (10 ng/ml; Sigma-Aldrich) and N-acetylcysteine (60 μg/ml; Sigma-Aldrich), and then plated at a density of 2 × 10^6^ cells/60 mm plate, as has been described before^32^.

### Western blot

Protein was extracted from the cultured cells with RIPA lysis buffer (1% NP40, 0.1% Sodium dodecyl sulfate (SDS), 100 μg/ml phenylmethylsulfonyl fluoride, 0.5% sodium deoxycholate, in PBS) on ice. The supernatants were collected after centrifugation at 12000 × g at 4 °C for 20 min. Protein concentration was determined using a BCA protein assay kit (Bio-rad, China), and whole lysates were mixed with 4 × SDS loading buffer (125 mmol/l Tris-HCl, 4% SDS, 20% glycerol, 100 mmol/l Dithiothreitol (DTT), and 0.2% bromophenol blue) at a ratio of 1:3. Samples were heated at 100 °C for 5 min and were separated on SDS-polyacrylamide gels. The separated proteins were then transferred to a PVDF membrane. The membrane blots were first probed with a primary antibody. After incubation with horseradish peroxidase-conjugated second antibody, autoradiograms were prepared using the enhanced chemiluminescent system to visualize the protein antigen. The signals were recorded using X-ray film. Primary antibodies were rabbit anti-β-catenin, anti-Beclin, anti-Atg7, anti-LC3 and anti-α-tubulin (Cell Signaling, San Jose, CA, USA). Secondary antibody is HRP-conjugated anti-rabbit (Jackson ImmunoResearch Labs, West Grove, PA, USA). α-tubulin was used as protein loading controls. The protein levels were first normalized to α-tubulin, and then normalized to control.

### Quantitative real-time PCR (RT-qPCR)

Total RNA were extracted from cultured cells with RNeasy kit (Qiagen), for cDNA synthesis. Quantitative real-time PCR (RT-qPCR) was performed in duplicates with QuantiTect SYBR Green PCR Kit (Qiagen). All primers were purchased from Qiagen. Data were collected and analyzed with 2−ΔΔCt method for quantification of the relative mRNA expression levels. Values of genes were first normalized against α-tubulin, and then compared to controls.

### Mouse manipulations

All mouse experiments were performed in accordance with the approved guidelines from the IACUC of Third Military Medical University. The methods were carried out in accordance with the approved guidelines. Twelve week-old female NOD/SCID mice (Jackson Lab, Bar Harbor, ME, USA) were used for experiments. Luciferase-carrying HCC cells (10^5^) were injected subcutaneously in the middle upper abdomen region to form tumor in 12 week-old female NOD/SCID mice. Four weeks later, GRh2 (1 mg/kg body weight) was injected from the tail vein of the mice twice per week for 4 weeks, till the end of experiment. Control mice received injection of saline of same volume and same frequency. Ten mice were used in each experimental group. Bioluminescence was measured with the IVIS imaging system (Xenogen Corp., Alameda, CA, USA). All of the images were taken 10 minutes after intraperitoneal injection of luciferin (Sigma-aldrich) of 150 mg/kg body weight, as a 60-second acquisition and 10 of binning. During image acquisition, mice were sedated continuously via inhalation of 3% isoflurane. Image analysis and bioluminescent quantification was performed using Living Image software (Xenogen Corp).

### Statistical analysis

All statistical analyses were carried out using the SPSS 18.0 statistical software package. All data were statistically analyzed using one-way ANOVA with a Bonferoni correction, followed by Fisher’s exact test. All values are depicted as mean ± standard deviation and are considered significant if p < 0.05.

## Results

### GRh2 dose-dependently inhibits HCC cell growth

**Figure 1:**
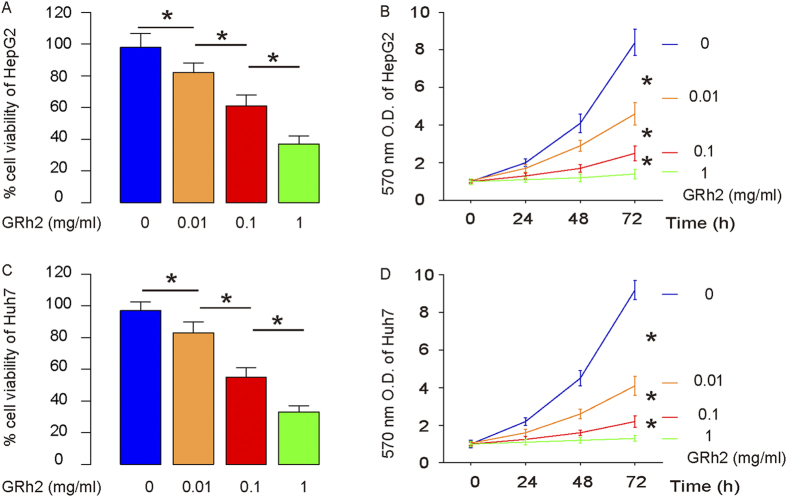
GRh2 dose-dependently inhibits HCC cell growth. We gave GRh2 at different doses (0.01 mg/ml, 0.1 mg/ml and 1 mg/ml) to the cultured HCC cells. (**A**,**B**) GRh2 dose-dependently decreased the cell viability of HepG2 cells in either a CCK-8 assay (**A**), or a MTT assay (**B**). (**C**,**D**) GRh2 dose-dependently decreased the cell viability of Huh7 cells in either a CCK-8 assay (**C**), or a MTT assay (**D**). *p < 0.05.

We examined the effect of GRh2 on the viability of HCC cells. We gave GRh2 at different doses (0.01 mg/ml, 0.1 mg/ml and 1 mg/ml) to 2 human HCC cell lines, HepG2 and Huh7. We found that from 0.01 mg/ml to 1 mg/ml, GRh2 dose-dependently decreased the cell viability of HepG2 cells in either a CCK-8 assay (Fig. 1A), or a MTT assay (Fig. 1B). Similarly, from 0.01 mg/ml to 1 mg/ml, GRh2 dose-dependently decreased the cell viability of Huh7 cells in either a CCK-8 assay (Fig. 1C), or a MTT assay (Fig. 1D). These data suggest that GRh2 dose-dependently inhibits HCC cell growth.

### GRh2 dose-dependently decreases CSCs-like cells in HCC cells

**Figure 2:**
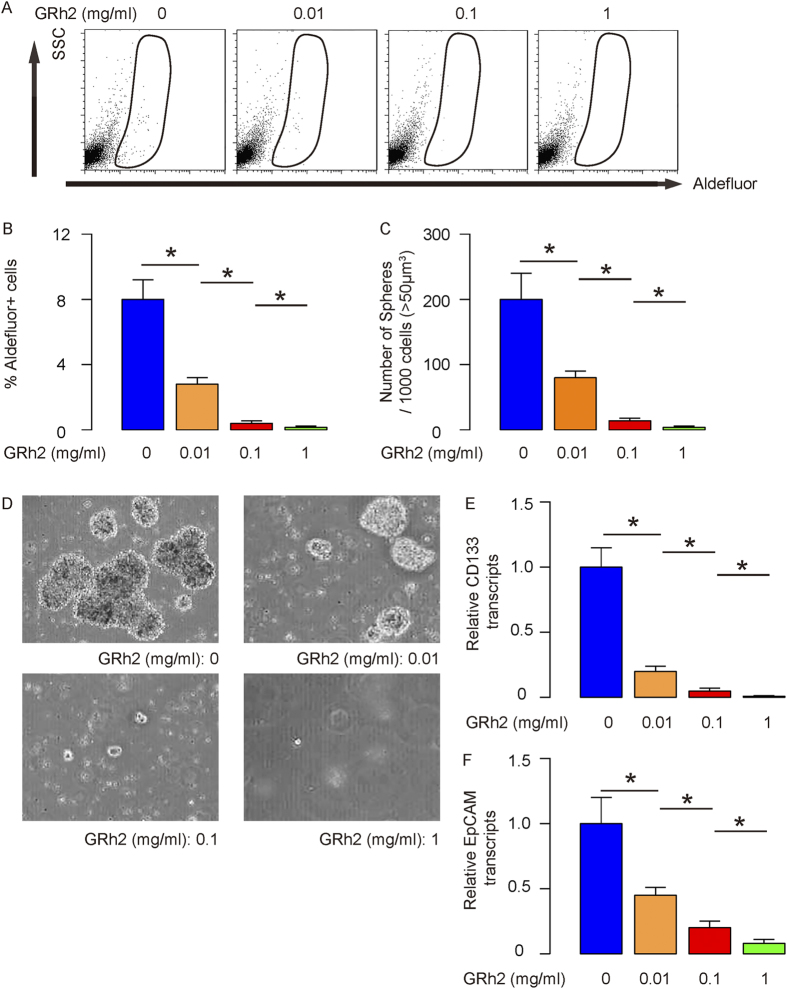
GRh2 dose-dependently decreases CSCs-like HepG2 cells. (**A**,**B**) GRh2 dose-dependently decreased the percentage of Aldefluor + cells, by representative flow charts (**A**), and by quantification (**B**). (**C**,**D**) GRh2 dose-dependently decreased the formation of tumor sphere-like structure, shown as quantification (**C**), and by representative images (**D**). (**E**,**F**) RT-qPCR for CD133 (**E**) and EpCAM (**F**). *p < 0.05. N = 5.

**Figure 3:**
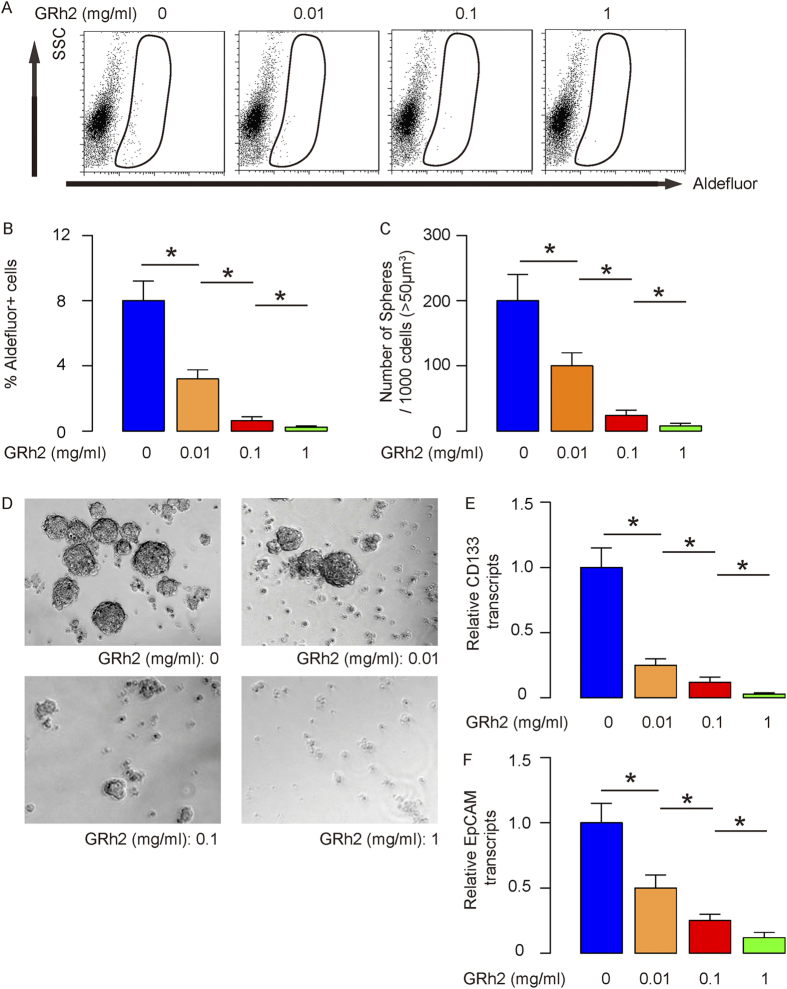
GRh2 dose-dependently decreases CSCs-like Huh7 cells. (**A**,**B**) GRh2 dose-dependently decreased the percentage of Aldefluor + cells, by representative flow charts (**A**), and by quantification (**B**). (**C**,**D**) GRh2 dose-dependently decreased the formation of tumor sphere-like structure, shown as quantification (**C**), and by representative images (**D**). (**E**,**F**) RT-qPCR for CD133 (**E**) and EpCAM (**F**). *p < 0.05. N = 5.

Next, we examined whether GRh2 treatment may affect CSCs-like cells. Thus, we analyzed the percentage of Aldefluor+ cells, which has been used to enrich CSCs cells. We found that GRh2 dose-dependently decreased the percentage of Aldefluor+ cells in HepG2 cells, shown by representative flow charts (Fig. 2A), and by data quantification (Fig. 2B). We then examined the capability of the GRh2-treated cells in the formation of tumor sphere, another feature for CSCs. We found that GRh2 dose-dependently decreased the formation of tumor sphere-like structure, shown by data quantification (Fig. 2C), and by representative images (Fig. 2D). Moreover, the levels of CSCs marker CD133 (Fig. 2E) and Epithelial cell adhesion molecule (EpCAM, Fig. 2F) were also dose-dependently suppressed by GRh2. These data were similarly reproduced in Huh7 cells (Fig. 3A–F). Together, these data suggest that GRh2 dose-dependently inhibits HCC cell growth, possibly through decreasing CSCs-like cells.

### GRh2 treatment inhibits growth of HCC cells *in vivo*

**Figure 4:**
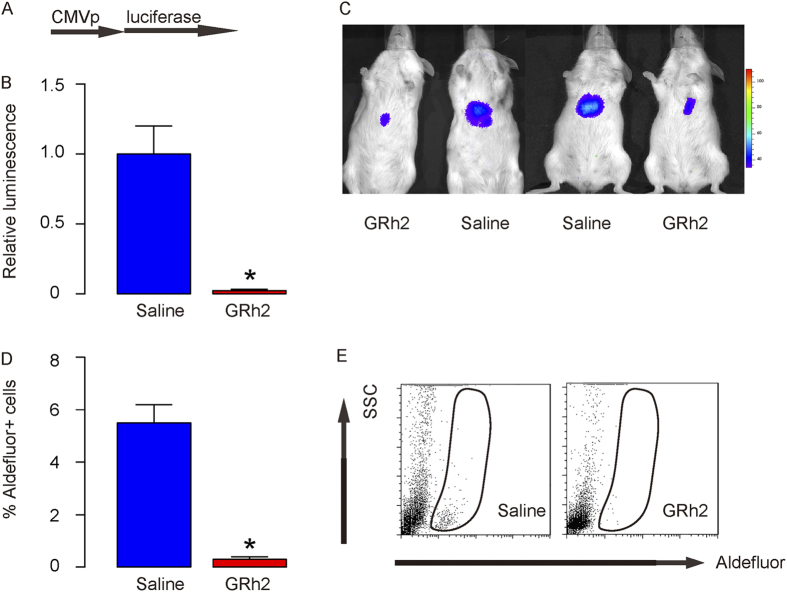
GRh2 treatment inhibits growth of HCC cells *in vivo*. (**A**) We transduced HepG2 cells with luciferease under a CMV promoter. (**B**,**C**) The transduced cells were used for *in vivo* tracing. We thus injected these cells subcutaneously into NOD/SCID mice to develop tumor. Four weeks later, GRh2 (1 mg/kg body weight) was injected from the tail vein of the mice twice per week for 4 weeks. Control mice received injection of saline of same volume and same frequency. Bioluminescence was then measured, showing significant impairment of implanted tumor growth by quantification (**B**), and by representative images (**C**). (**D**,**E**) The dissected and dissociated tumor from mice treated with GRh2 had significantly lower percentage of Aldefluor + cells, shown by quantification (**D**), and by representative flow charts (**E**). *p < 0.05. Each experiment group contained 10 mice.

In order to figure out whether GRh2 similarly inhibits growth of HCC *in vivo*, we transduced HepG2 cells with luciferease under a CMV promoter (Fig. 4A). The transduced cells were used for *in vivo* tracing. We thus injected these cells subcutaneously into NOD/SCID mice to develop tumor. Four weeks later, GRh2 (1 mg/kg body weight) was injected from the tail vein of the mice twice per week for 4 weeks. Control mice received injection of saline of same volume and same frequency. Bioluminescence was then measured, showing significant impairment of implanted tumor growth by quantification (Fig. 4B), and by representative images (Fig. 4C). Moreover, the dissected and dissociated tumor from mice treated with GRh2 had significantly lower percentage of Aldefluor+ cells (Fig. 4D,E), suggesting that GRh2 treatment decreases CSCs of HCC and inhibits growth of HCC cells *in vivo*.

### GRh2 treatment decreases β-catenin and increases autophagy in HCC cells

**Figure 5:**
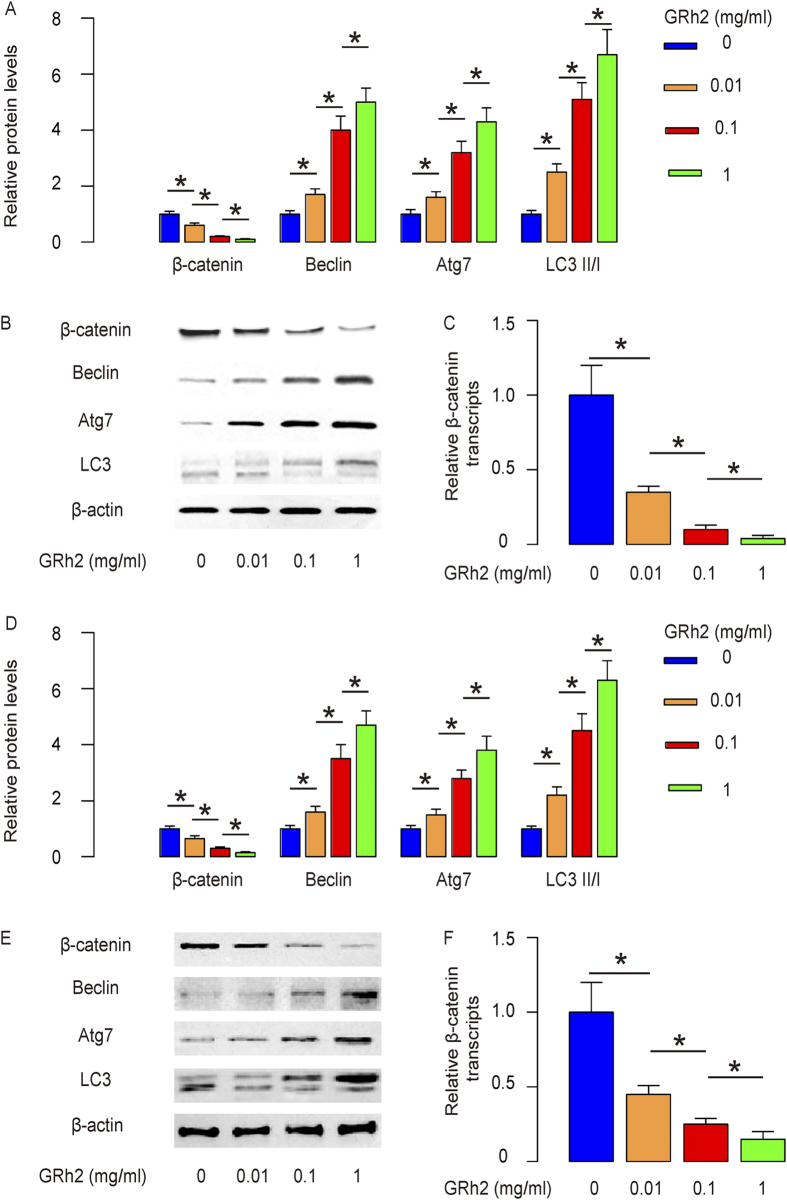
GRh2 treatment decreases β-catenin and increases autophagy in HCC cells. (**A**,**B**) GRh2 treatment dose-dependently decreases β-catenin, and dose-dependently upregulated autophagy-related proteins Beclin, Atg7 and increased the ratio of LC3 II to LC3 I, by quantification (**A**), and by representative Western blots (**B**) in HepG2 cells. (**C**) GRh2 treatment dose-dependently decreases β-catenin transcripts in HepG2 cells. (**D**,**E**) GRh2 treatment dose-dependently decreases β-catenin, and dose-dependently upregulated autophagy-related proteins Beclin, Atg7 and increased the ratio of LC3 II to LC3 I, shown by quantification (**E**), and by representative Western blots (**F**) in Huh7 cells. (**G**) GRh2 treatment dose-dependently decreases β-catenin transcripts in Huh7 cells. *p < 0.05. N = 5.

We thus studied the molecular mechanisms underlying the cancer inhibitory effects of GRh2 on HCC cells. We examined the growth-regulatory proteins in HCC. From a variety of proteins, we found that GRh2 treatment dose-dependently decreased β-catenin, and dose-dependently upregulated autophagy-related proteins Beclin, Atg7 and increased the ratio of LC3 II to LC3 I in HegG2 cells, shown by quantification (Fig. 5A), and by representative Western blots (Fig. 5B). Moreover, the dose-dependent inhibition of β-catenin by GRh2 was also detected at transcription level (Fig. 5C). These data were similarly reproduced in Huh7 cells (Fig. 5D–F). Since β-catenin signaling is a strong cell-growth stimulator and autophagy can usually lead to stop of cell-growth and cell death^33^,^34^,^35^,^36^,^37^, we feel that the alteration in these pathways may be responsible for the GRh2-mediated suppression of HCC growth.

### Inhibition of autophagy abolishes the effects of GRh2 on β-catenin

**Figure 6:**
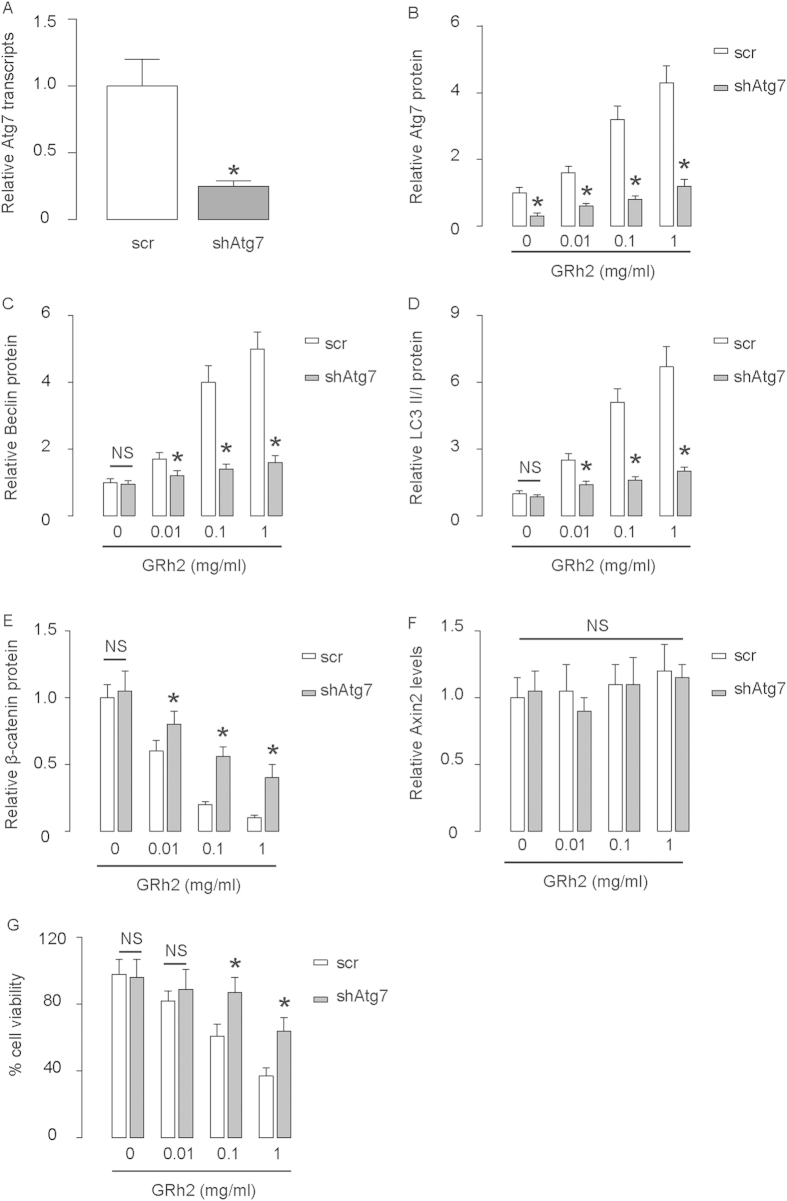
Inhibition of autophagy in HepG2 cells abolishes the effects of GRh2 on β-catenin. HepG2 cells were transfected with shRNA for Atg7, or scrambled sequence (scr) as a control. (**A**) RT-qPCR for Atg7. (**B**–**E**) Western blot for Atg7 (**B**), Beclin (**C**), LC3 (**D**) and β-catenin (**E**). (**F**) RT-qPCR for Axin2. (**G**) Cell viability by CCK-8 assay. *p < 0.05. NS: non-significant. N = 5.

**Figure 7:**
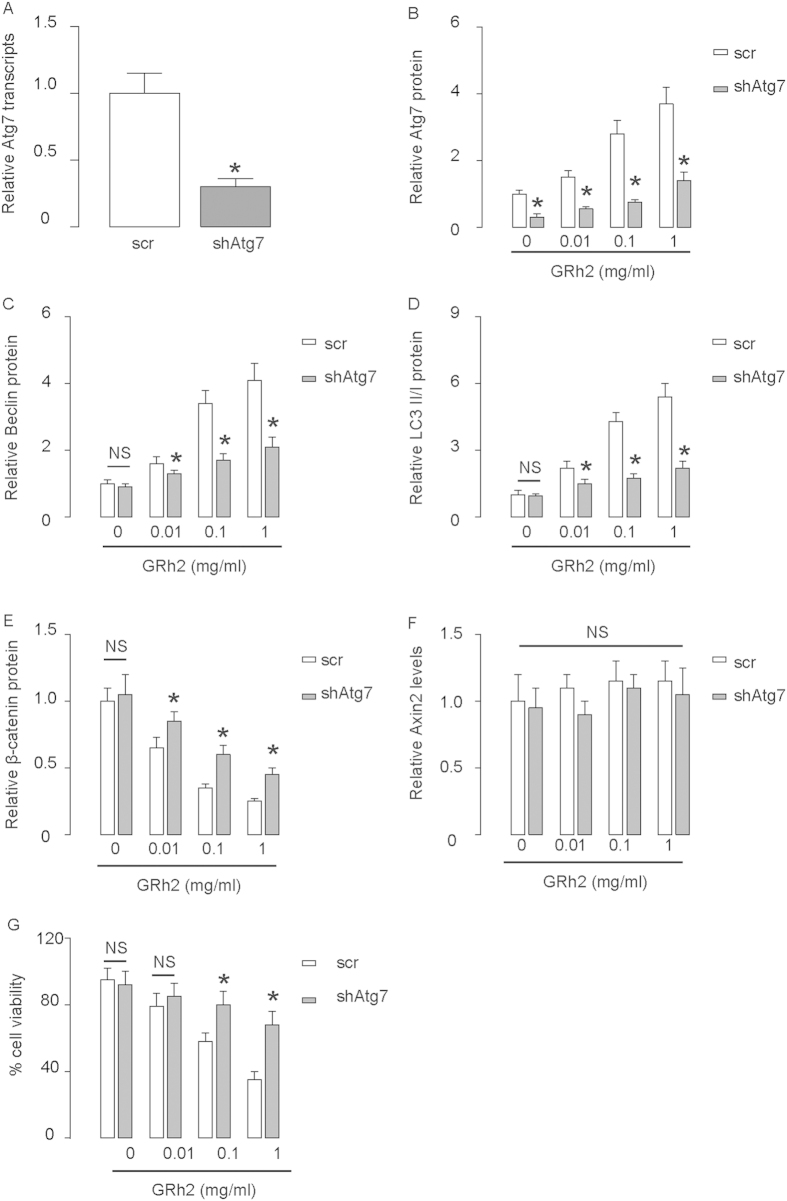
Inhibition of autophagy in Huh7 cells abolishes the effects of GRh2 on β-catenin. Huh7 cells were transfected with shRNA for Atg7, or scrambled sequence (scr) as a control. (**A**) RT-qPCR for Atg7. (**B–E**) Western blot for Atg7 (**B**), Beclin (**C**), LC3 (**D**) and β-catenin (**E**). (**F**) RT-qPCR for Axin2. (**G**) Cell viability by CCK-8 assay. *p < 0.05. NS: non-significant. N = 5.

In order to find out the relationship between β-catenin and autophagy in this model, we inhibited autophagy using a shRNA for Atg7, and examined its effect on the changes of β-catenin by GRh2. First, the inhibition of Atg7 in HepG2 cells by shAtg7 was confirmed by RT-qPCR (Fig. 6A), and by Western blot (Fig. 6B). Inhibition of Atg7 resulted in abolishment of the dose-dependent effects of GRh2 on other autophagy-associated proteins (Fig. 6C,D), and resulted in abolishment of the inhibitory effect of GRh2 on β-catenin (Fig. 6E), without affecting Axin2 levels (Fig. 6F). Moreover, the effects of GRh2 on cell viability were significantly inhibited (Fig. 6G). These data were similarly reproduced in Huh7 cells (Fig. 7A–G). Together, our data suggest that inhibition of autophagy abolishes the effects of GRh2 on β-catenin. Thus, the regulation of GRh2 on β-catenin needs autophagy-associated proteins.

### Overexpression of β-catenin abolishes the effects of GRh2 on autophagy

**Figure 8:**
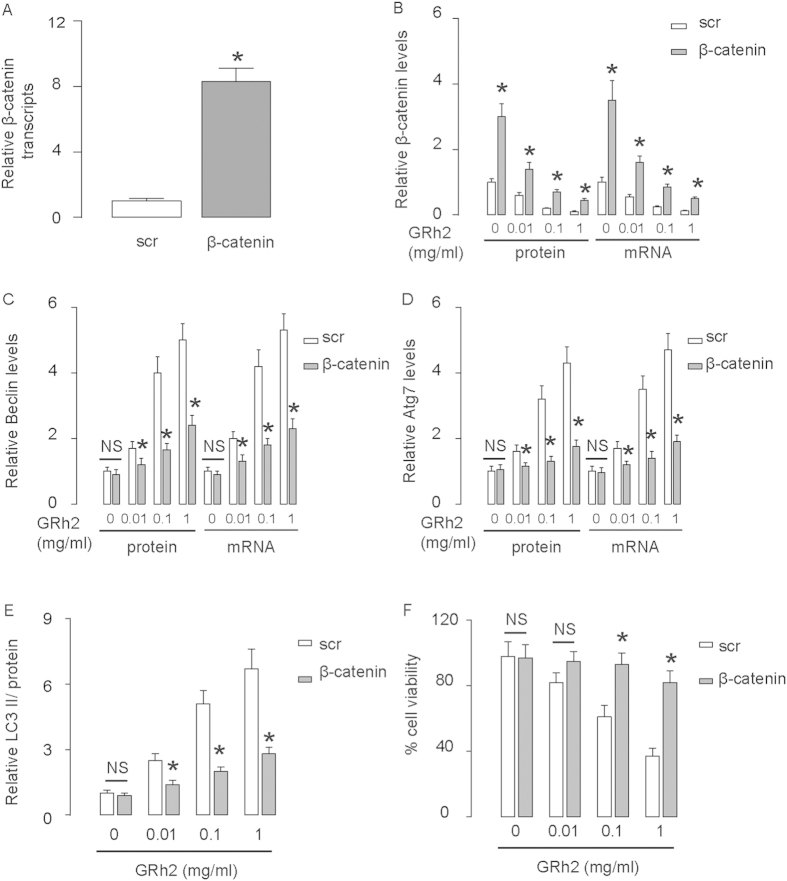
Overexpression of β-catenin in HepG2 cells abolishes the effects of GRh2 on autophagy. HepG2 cells were transfected with β-catenin, or scrambled sequence (scr) as a control. (**A**) RT-qPCR for β-catenin. (**B**–**E**) Western blot β-catenin (**B**), Beclin (**C**), Atg7 (**D**) and LC3 (**E**). (**F**) Cell viability by CCK-8 assay. *p < 0.05. NS: non-significant. N = 5.

**Figure 9:**
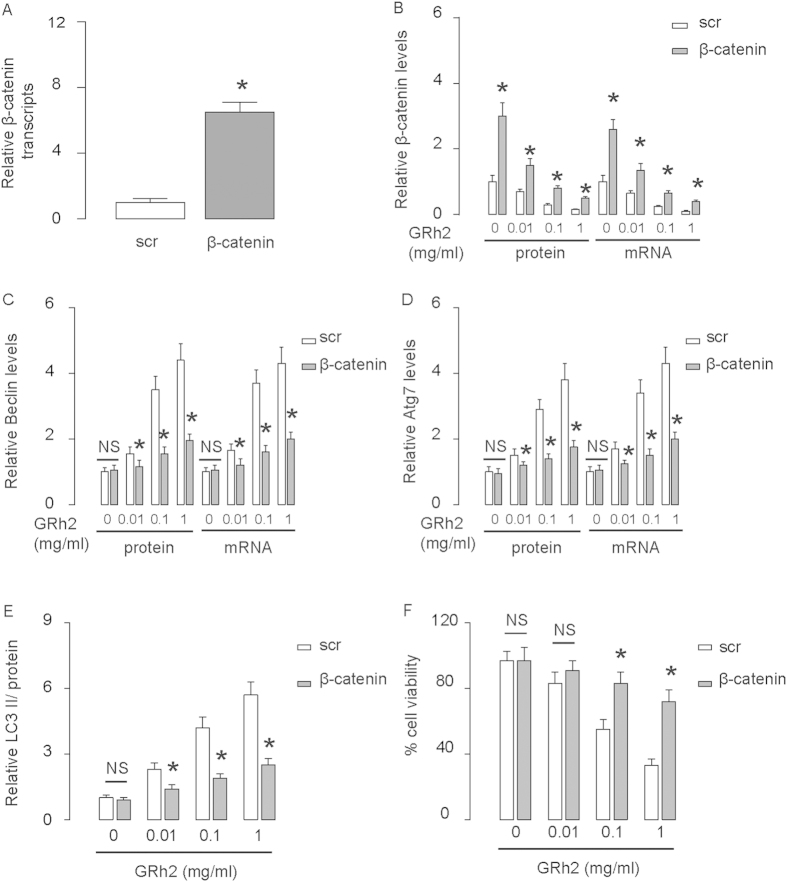
Overexpression of β-catenin in Huh7 cells abolishes the effects of GRh2 on autophagy. Huh7 cells were transfected with β-catenin, or scrambled sequence (scr) as a control. (**A**) RT-qPCR for β-catenin. (**B**–**E**) Western blot β-catenin (**B**), Beclin (**C**), Atg7 (**D**) and LC3 (**E**). (**F**) Cell viability by CCK-8 assay. *p < 0.05. NS: non-significant. N = 5.

**Figure 10:**
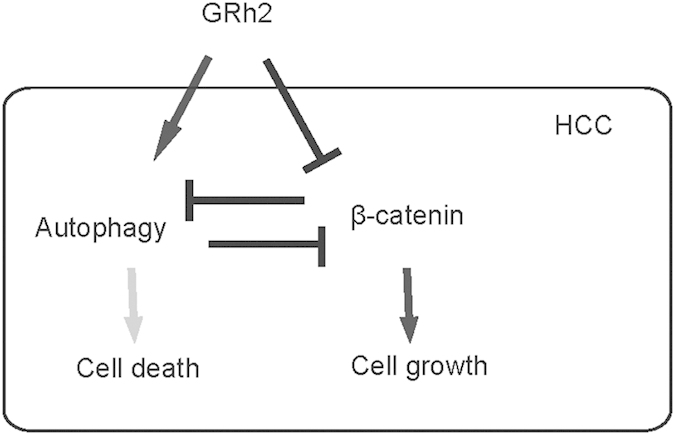
Schematic of the model. GRh2 may target both β-catenin signaling and autophagy, which interacts with each other in the regulation of HCC cell viability and growth. Autophagy could progress into cell death, when the machinery runs out of its limitation.

Next, we inhibited the effects of GRh2 on β-catenin by overexpression of β-catenin in HepG2 cells. First, the overexpression of β-catenin in HepG2 cells was confirmed by RT-qPCR and by Western blot (Fig. 8A,B). Overexpression of β-catenin resulted in abolishment of the dose-dependent effects of GRh2 on autophagy-associated proteins (Fig. 8C–E). Moreover, the effects of GRh2 on cell viability were significantly inhibited (Fig. 8F). These data were similarly reproduced in Huh7 cells (Fig. 9A–F). Thus, inhibition of β-catenin signaling abolishes the effects of GRh2 on autophagy, and the regulation of GRh2 on autophagy needs β-catenin signaling. This model is summarized in a schematic (Fig. 10), showing that GRh2 may target both β-catenin signaling and autophagy, which interact with each other to regulate HCC cell viability and growth. Autophagy could progress into cell death (autophagic cell death), when the cell survival machinery runs out of its limitation^33^,^34^,^35^,^36^,^37^.

## Discussion

In the current study, we analyzed the effects of GRh2 on the viability of HCC. Importantly, we not only found that GRh2 dose-dependently decreases HCC cell viability, but also dose-dependently decreased the number of Aldefluor+ CSCs-like in HCC cells. These data suggest that the CSCs-like cells in HCC may be more susceptible for the GRh2 treatment, and the decreases in CSCs-like cells may result in the decreased viability in total HCC cells. This point was supported by following studies on mechanisms. It has been well known that activated β-catenin signaling by WNT/GSK3β prevents degradation of β-catenin and induces its nuclear translocation^38^. Nuclear β-catenin activates c-myc, cyclinD1 and c-jun to promote cell proliferation, and activates Bcl-2 to inhibit apoptosis^38^. High β-catenin levels thus may be a signature of CSCs-like cells. Therefore, it is not surprising that CSCs-like cells are more susceptible than other non-CSCs when GRh2 is applied. Interestingly, modulation of β-catenin levels by Atg7 in HCC cells did not activate Axin2^39^,^40^, the negative regulator of β-catenin, possibly as a distinguished manner in this crosstalk between autophagy and β-catenin signaling pathway.

In addition, GRh2 appears to target autophagy. Although altered metabolism may be beneficial to the cancer cells, it can create an increased demand for nutrients to support cell growth and proliferation, which creates metabolic stress and subsequently induces autophagy, a catabolic process leading to degradation of cellular components through the lysosomal system^41^. Cancer cells use autophagy as a survival strategy to provide essential biomolecules that are required for cell viability under metabolic stress^41^. However, autophagy not only results in a staring in cell growth, but also may result in cell death^33^,^34^,^35^,^36^,^37^,^41^. Increases in autophagy may substantially decrease cancer cell growth. Thus, GRh2 has its inhibitory effect on HCC cells through a combined effect on cell proliferation (by decreasing β-catenin) and autophagy^41^.

Interestingly, our data suggest an interaction between β-catenin and autophagy. This finding is consistent with previous reports showing that autophagy negatively modulates Wnt/β-catenin signaling by promoting Dvl instability^42^,^43^, and with other studies showing that β-catenin regulates autophagy^44^,^45^,^46^.

Of note, we have used 2 HCC lines and essentially got same results. Hence, these findings suggest a promising GRh2 therapy, which could be performed in a sufficiently frequent manner, to substantially improve the current treatment for HCC.

## Additional Information

**How to cite this article**: Yang, Z. *et al*. Ginsenoside Rh2 inhibits hepatocellular carcinoma through ß-catenin and autophagy. *Sci. Rep*. **6**, 19383; doi: 10.1038/srep19383 (2016).
